# General Anesthesia Surgery for Early Breast Cancer in a Patient with Severe Heart Failure due to Dilated Cardiomyopathy: A Case Report

**DOI:** 10.70352/scrj.cr.25-0034

**Published:** 2025-04-09

**Authors:** Tomohiro Oshino, Karin Shikishima, Yumi Moriya, Mitsuchika Hosoda, Kiwamu Kamiya, Toshiyuki Nagai, Toshihisa Anzai, Masato Takahashi

**Affiliations:** 1 Department of Breast Surgery, Hokkaido University Hospital, Sapporo, Hokkaido, Japan; 2 Department of Cardiovascular Medicine, Faculty of Medicine and Graduate School of Medicine, Hokkaido University, Sapporo, Hokkaido, Japan

**Keywords:** breast cancer surgery, dilated cardiomyopathy, severe heart failure, left ventricular ejection fraction

## Abstract

**INTRODUCTION:**

Perioperative mortality is significantly higher in cases of heart failure with severe left ventricular ejection fraction (LVEF) reduction, making it challenging to decide whether to proceed with surgery for early-stage breast cancer, which is not immediately fatal. However, the prognosis of heart failure has improved and breast cancer is increasingly becoming a prognostic factor. Herein, we report the case of a breast cancer patient with severe heart failure due to dilated cardiomyopathy (DC), who was deemed fit to undergo surgery under general anesthesia after obtaining sufficient informed consent and achieving improvement in heart failure symptoms during endocrine therapy.

**CASE PRESENTATION:**

A 64-year-old female with a history of DC and sustained ventricular tachycardia, who had received cardiac resynchronization therapy with defibrillator implantation, underwent breast cancer surgery. She had been repeatedly hospitalized for heart failure with an LVEF of 19% and New York Heart Association (NYHA) Class III status, and heart transplant surgery was considered. However, a screening computed tomography scan revealed right breast cancer, and neither heart transplantation nor breast cancer surgery was performed. Endocrine therapy was initiated and failed 48 months after administration. Although the LVEF remained low at 21%, the NYHA classification improved to Class II, and she had not been hospitalized for heart failure for an extended period since her breast cancer diagnosis. Therefore, breast cancer surgery was performed under general anesthesia and no postoperative complications were observed throughout the course of the surgery.

**CONCLUSION:**

Given that the prognosis for heart failure may statistically be better than that for breast cancer, early breast cancer surgery should be performed in patients with stable heart failure symptoms.

## Abbreviations

CDK
: cyclin-dependent kinase
CRT-D
: cardiac resynchronization therapy defibrillator
CT
: computed tomography
DC
: dilated cardiomyopathy
ER
: estrogen receptor
HER2
: human epidermal growth factor receptor type 2
LVEF
: left ventricle ejection fraction
MAGGIC score
: Meta-analysis Global Group in Chronic Heart Failure
NYHA
: New York Heart Association

## INTRODUCTION

Although drug therapy for early-stage breast cancer has been developing rapidly,^1,2)^ surgery remains essential. Furthermore, patients with breast cancer often have a medical history that includes conditions such as heart disease.

Perioperative mortality has been reported to be significantly increased in cases of heart failure with severe left ventricular ejection fraction (LVEF) reduction due to heart diseases like dilated cardiomyopathy (DC). When the LVEF decreases to less than 40%, the risk of developing perioperative cardiovascular complications (death, acute heart failure, and acute myocardial infarction),^3)^ as well as the 90-day mortality rate, increases.^4)^ Furthermore, an LVEF of <30% is an independent determinant of mortality, not only during the perioperative period, but also in the long term after surgery.^5)^ Therefore, when a patient has severe heart failure with a low LVEF, it can be challenging to decide whether to perform surgery for early breast cancer, which is not immediately fatal. Although treatment is chosen based on a comparison of risks and benefits, there are almost no reports of general anesthesia surgery in patients with early breast cancer and severely reduced LVEF. However, in recent years, improvements in the prognoses of DC^6)^ and heart failure,^7)^ have increased the significance of breast cancer as a prognostic factor.

We report the case of a breast cancer patient with severe heart failure due to DC, who showed improvement in heart failure symptoms during endocrine therapy but eventually underwent surgery after the failure of endocrine therapy.

## CASE PRESENTATION

A 64-year-old female presented with breast cancer, having a body mass index of 26 kg/m^2^, and a systolic blood pressure of 98 mmHg. She had a history of dilated cardiomyopathy, which developed at the age of 43, sustained ventricular tachycardia, and had undergone cardiac resynchronization therapy defibrillator (CRT-D) implantation. Her medical history also included chronic kidney disease G4 with a serum creatinine level of 2.44 mg/dL and controlled diabetes mellitus. Her medications included bisoprolol fumarate, pimobendan, eplerenone, losartan potassium, furosemide, amiodarone hydrochloride, warfarin potassium, and insulin.

**Fig. 1:**
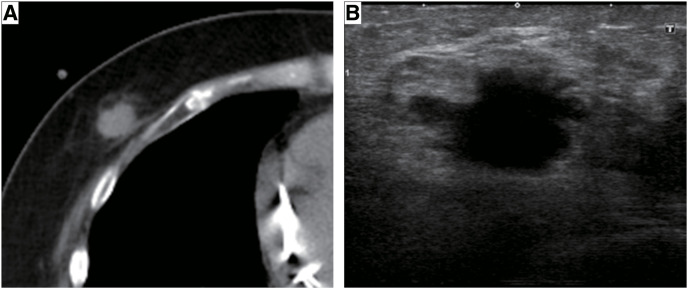
Imaging of breast cancer at diagnosis. (A) Plain computed tomography imaging showed right breast tumor. (B) Ultrasonography imaging showed hypoechoic 19 mm mass.

**Fig. 2:**
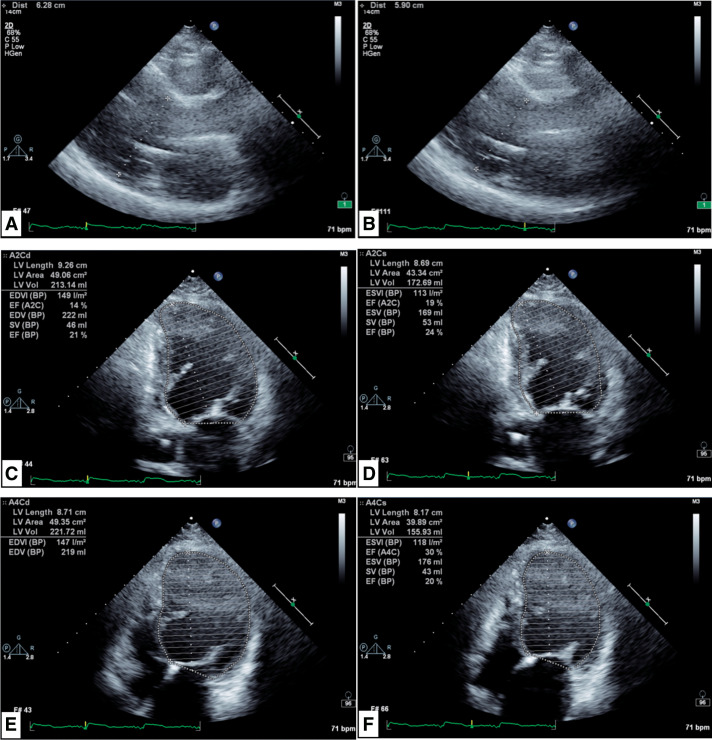
Cardiac echo imaging before breast cancer surgery. (A) The left ventricular diameter during diastole was 63 mm. (B) Left ventricular diameter during systole was 59 mm. (C)–(F) Modified Simpson method measured left ventricle ejection fraction to be 21%.

**Table 1** shows the time course of DC and breast cancer. The patient was repeatedly admitted to the hospital due to heart failure, and heart transplant surgery was considered. However, a right breast tumor was detected upon plain computed tomography (CT) scan imaging (**Fig. 1**). She was diagnosed with invasive ductal carcinoma, positive estrogen receptor (ER) (100%), human epidermal growth factor receptor type 2 (HER2) score of 1, Ki-67 index of 23.1%, and T1c (19 mm) N1M0 Stage 2A. Therefore, she was deemed inappropriate for a heart transplant. Additionally, she had severe heart failure with an LVEF of 19% and New York Heart Association (NYHA) Class III status, and was deemed unable to undergo surgery under general anesthesia. Therefore, she was prescribed anastrozole. Although anastrozole was effective, it was discontinued after 9 months due to the need for a battery replacement for the CRT-D. After 14 months of administration, percutaneous mitral valve repair (MitraClip®, Abbott) was performed under general anesthesia for severe functional mitral regurgitation, and improvement in heart failure was observed, with the NYHA classification improving to Class II. At this point, the risks of general anesthesia for breast cancer surgery could not be ignored, and endocrine therapy was continued without surgery. As the tumor had grown, anastrozole was switched to tamoxifen, which suppressed the breast cancer growth. After 19 months of tamoxifen administration, tumor growth was observed and anastrozole was resumed. However, 15 months after anastrozole administration, tumor growth was observed again. As there were no additional endocrine therapies for perioperative treatment, the indications for surgery were reconsidered. Although the LVEF was still low at 21% (**Fig. 2**), the NHYA classification had improved to Class II, and she had not been hospitalized for heart failure for a long time. Therefore, we decided to perform the surgery if she wished, after thoroughly explaining the risks outlined in the consent form, which included risks specific to her case (**Table 2**), with an intensive care unit (ICU) backup.

**Table 1:** Patient case history

Year	Events for heart disease	LVEF, NYHA, MR or heart failure situation	Breast cancer
X − 19	Diagnosis of DC	LVEF 23%	
X − 17	ICD implantation for non-sustained VT		
X − 11	ICD generator Replacement	LVEF 38%	
X − 5	CRT-D implantation for DC	Acute bronchitis led to decompensated heart failure → compensated with drug therapy. LVEF 26%, MR moderate	
X − 1		Repeatedly admitted to and discharged from hospital due to uncontrolled heart failure	
X		LVEF 18%, NYHA 3, MR severe, consideration of a heart transplant	Right breast cancer T1c (19 mm) N1M0 ANA administration
X + 1	CRT-D battery replacement	LVEF 20%, NYHA 3, MR severe	Discontinuation of ANA Tumor size 8.4 mm
X + 2	Mitral valve replacement surgery	LVEF 22%, NYHA 2S, MR mild	TAM administration Tumor size 14 mm
X + 3		LVEF 21%	Change to ANA Tumor size 23 mm
X + 4		LVEF 21%, NYHA 2S, MR mild	Progression disease Right.Bt+Ax

LVEF, left ventricular ejection fraction; NYHA, New York Heart Association; MR, mitral regurgitation; DC, dilated cardiomyopathy; ICD, implantable cardioverter defibrillator, VT, ventricular tachycardia; CRT-D, cardiac resynchronization therapy defibrillator; ANA, Anastrozole, TAM, Tamoxifen, Bt, total mastectomy; Ax, axillary dissection.

**Table 2:** Additional explanations for informed consent

Explanation
**Any cardiovascular event (10%)**
Death
Progression of heart failure
Shock
Arrhythmia (including fatal ventricular arrhythmia)
Myocardial infarction
Renal failure, introduction of hemodialysis
Infective endocarditis
**Breast cancer recurrence**

The patient was admitted to the hospital, warfarin was replaced with heparin, and a right total mastectomy with axillary dissection (levels I + II) was performed. Anesthesia duration was 3 h 42 min, surgery duration was 2 h 24 min, with 850 mL of crystalloid solution infusion, urine volume of 600 mL, and blood loss of 120 mL. Hemodynamic stability was achieved without the use of catecholamines, with a continuous intravenous infusion of phenylephrine and administration of ephedrine when necessary, along with continuous arterial blood pressure monitoring. The patient was admitted to the ICU after surgery and was discharged from the general ward the following day. Fluid volume was assessed based on urine volume, body weight, and chest radiography findings. No postoperative complications were observed throughout the course of the surgery.

**Fig. 3:**
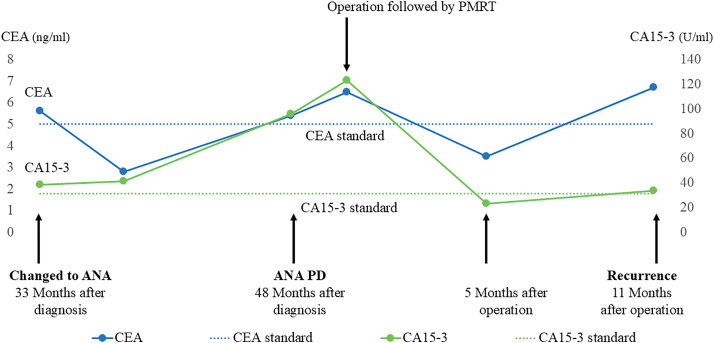
Changes in tumor markers. ANA, anastrozole; PD, progressive disease; PMRT, postmastectomy radiation therapy; CEA, carcinoembryonic antigen; CA15-3, cancer antigen 15-3

Postoperative pathology revealed invasive micropapillary carcinoma, ypT1c (19 mm), ypN2a (5/10), histological grade 3, ER 100%, HER2 score of 1, and a Ki-67 index of 26.0%. As no pathogenic variants of *BRCA* were detected and it was deemed that chemotherapy and abemaciclib would be difficult to administer, only exemestane was initiated as postoperative drug therapy. Postoperative radiation therapy was administered to the chest wall and supraclavicular lymph node region (50 Gy/25 Fr) to avoid radiation to the heart. 5 months after the operation, the tumor marker levels decreased to within the normal range; however, 11 months post-operation, they increased again (**Fig. 3**). The CT scan revealed metastasis in the lymph nodes dorsal to the pectoralis muscle. Treatment for recurrent breast cancer with fulvestrant alone was initiated.

## DISCUSSION

In this case, we were able to perform breast cancer surgery without any complications, considering the LVEF, the long-term stability of heart failure symptoms, and improvement in NYHA classification. However, local recurrence occurred, suggesting that early breast cancer may be a more significant prognostic factor than heart failure.

Appropriate anesthesia and perioperative management are crucial, as perioperative cardiovascular events increase with reduced LVEF in heart failure. The reasons for this include myocardial damage caused by stress hormones produced during surgery,^8,9)^ as well as a transient state of excessive imbalance caused by general anesthesia and the administration of replacement fluids.^10)^ In this case, we limited the amount of postoperative fluid administered and no postoperative complications occurred. In a report on 13 colorectal cancer patients with severe chronic heart failure who underwent surgery under general anesthesia,^11)^ continuous arterial blood pressure monitoring was performed in all cases. Unlike this case, 7 patients required catecholamines to maintain hemodynamics. Therefore, general anesthesia should be administered with careful hemodynamic management.

In contrast, breast cancer surgery in patients with severe heart failure appears to be rarely performed. A PubMed search using the keywords ‘breast cancer’ and ‘heart failure,’ ‘dilated cardiomyopathy,’ or ‘left ventricular ejection fraction’ yielded no relevant case reports.

Predicting prognosis in patients with chronic heart failure or breast cancer who have not undergone surgery can be challenging. The Meta-analysis Global Group in Chronic (MAGGIC) Heart Failure score,^12)^ a prognostic score for chronic heart failure, was 30 at the time of the patient’s breast cancer diagnosis, and the estimated 3-year mortality rate was 12%.^13)^ However, the prognosis of heart failure,^7)^ as well as that of patients with severe heart failure who have undergone CRT-D implantation^6)^ and patients with DC who have not undergone heart transplantation, has improved. On the other hand, the effective duration of endocrine therapy for non-surgical treatment of ER+HER2- breast cancer is 1.6 years (range 0.5–7.0 years).^14)^ In patients with early breast cancer and heart failure, early breast cancer may emerge as a prognostic factor, even if endocrine therapy is administered. In the present case, endocrine therapy did not suppress the progression of breast cancer in the long term, and surgery was performed when both aromatase inhibitors and tamoxifen failed. Although surgery was performed, local recurrence occurred soon after. If breast surgery had been performed when heart failure symptoms improved, the breast cancer might not have recurred. However, at that point, deciding on surgery might have been difficult for the patient. Still, assessing the risks of heart disease and breast cancer and informing the patient of surgical options based on their progression would have been essential, beginning at the time of diagnosis.

The prognosis for ER+HER2-breast cancer that has recurred early has also improved in recent years owing to CDK4/6 inhibitors.^15,16)^ However, in cases of heart failure, renal failure may also be present, as in this case, where the safety of CDK4/6 inhibitors has not been confirmed, leading to the avoidance of these drugs. As a result, the expected long-term prognosis for ER+HER2-recurrent breast cancer may not be achieved.

## CONCLUSION

A patient with heart failure and severely reduced LVEF due to DC and breast cancer underwent general anesthesia surgery with appropriate cardiac function evaluation and sufficient informed consent. Considering that the prognosis of DC and heart failure may be better than that of breast cancer, early breast cancer surgery should be performed if heart failure symptoms are stable.

## ACKNOWLEDGMENTS

The authors thank Editage (www.editage.jp) for English language editing.

## DECLARATIONS

### Funding

Not applicable.

### Authors’ contributions

TO, KS, YM, MH, and MT were involved in patient care and surgery.

KK, TN, and TA were involved in the treatment and evaluation of cardiovascular diseases.

TO prepared the manuscript.

All authors have read and approved the manuscript.

### Availability of data and materials

Not applicable

### Ethics approval and consent to participate

Not applicable.

### Consent for publication

Written informed consent was obtained from the patient for the publication of this case report.

### Competing interests

MT received honoraria from Astra Zeneca, Eisai, Eli Lilly, Daiichi Sankyo, and MSD.

Other authors have no conflicts of interest.
